# Co-administration of combretastatin A4 nanoparticles and anti-PD-L1 for synergistic therapy of hepatocellular carcinoma

**DOI:** 10.1186/s12951-021-00865-w

**Published:** 2021-05-01

**Authors:** Bonan Zhao, Zhipeng Dong, Weixing Liu, Fangning Lou, Qiyan Wang, Hao Hong, Yue Wang

**Affiliations:** 1 Key Laboratory of Biomedical Functional Materials, School of Sciences, China Pharmaceutical University, 639 Longmian Avenue, Nanjing, 211198 China; 2 School of Medicine, Nanjing University, 22 Hankou Road, Nanjing, 210093 China

**Keywords:** Combretastatin A4(CA4), Nanoparticles, Anti-PD-L1(aPD-L1), Synergistic therapy

## Abstract

**Background:**

According to data estimated by the WHO, primary liver cancer is currently the fourth most common malignant tumor and the second leading cause of death around the world. Hepatocellular carcinoma (HCC) is one of the most common primary liver malignancies, so effective therapy is highly desired for HCC.

**Results:**

In this study, the use of poly(l-Aspartic acid)-poly(ethylene glycol)/combretastatin A4 (CA4-NPs) was aimed to significantly disrupt new blood vessels in tumor tissues for targeted hepatic tumor therapy. Here, PEG-b-PAsp-g-CA4 showed significantly prolonged retention in plasma and tumor tissue. Most importantly, CA4-NPs were mainly distributed at the tumor site because of the triple target effects—enhanced permeability and retention (EPR) effect, acid-sensitive (pH = 5.5) effect to the tumor microenvironment (TME), and good selectivity of CA4 for central tumor blood vessel. Considering that CA4-NPs might induce severe hypoxic conditions resulting in high expression of HIF-1α in tumor tissues, which could induce the overexpression of PD-L1, herein we also used a programmed death-ligand 1 antibody (aPD-L1) to prevent immunosuppression. This way of complementary combination is able to achieve an ideal treatment effect in tumor site where CA4-NPs and aPD-L1 could respond to the inner area and peripheral area, respectively. As a result, a significant decrease in tumor volume and weight was observed in the combination group of CA4-NPs plus aPD-L1 compared with CA4-NPs or aPD-L1 monotherapy in subcutaneous Hepa1-6 hepatic tumor models.

**Conclusions:**

We presented a new idea that co-administration of CA4-NPs and aPD-L1 possessed notable anti-tumor efficacy for HCC treatment.

**Graphic abstract:**

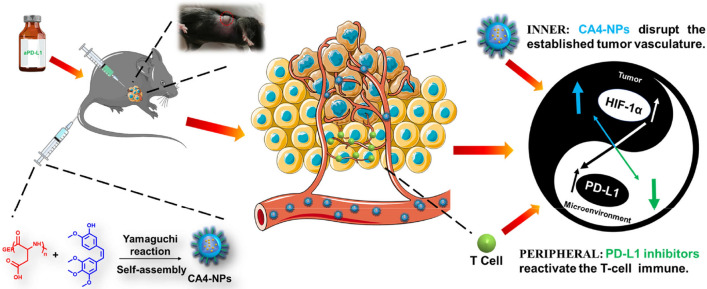

**Supplementary Information:**

The online version contains supplementary material available at 10.1186/s12951-021-00865-w.

## Introduction

Hepatocellular carcinoma (HCC) is the second leading cause of cancer-related death worldwide and more than half of the new cases and deaths occurred in Asia [1, 2]. As it is challenging to diagnose HCC early, a principal therapeutic challenge is the management of its highly malignant features and rapid progression. Due to the low rate of early diagnosis and rapid disease progression, the mortality rate of HCC remains high [3]. Surgery and liver transplantation are the best treatments for patients with liver cancer while the clinical treatment of advanced unresectable liver cancer remains a challenge. Therefore, for patients with unresectable hepatocellular carcinoma, transarterial chemoembolization (TACE) is recommended. However, TACE is a local treatment method, which is not effective in treating large tumors and advanced liver cancer [4–6]. The concurrent side effects also limit the application of TACE such as liver failure and gastrointestinal bleeding, etc. [7, 8]. But a large amount of clinical data showed that the use of traditional chemotherapy drugs to replace TACE did not bring satisfactory anti-tumor effects, so the development of new effective drugs against HCC has important clinical significance.

Vascular disrupting agents (VDAs) differ from traditional chemotherapy drugs in that they can not only eliminate the immature blood vessels in tumors preferentially but also modulate the tumor microenvironment (TME) selectively [9–11]. Combretastatin A4 phosphate (CA4P), as a representative VDA, can cause significant central tumor necrosis while leaving a thin layer of viable tumor cells at the tumor periphery [12, 13]. CA4 itself has high selectivity for formed tumor blood vessels. Actually, a critical point in improving the therapeutic efficacy of CA4 further is to keep a constant concentration around the endothelial cells and enhance the action time on tubulin. Nanocarrier-based drug delivery systems provide an ideal platform to work out. Nanocarrier-enwrapped or nanocarrier-conjugated drugs have the advantage of long-term circulation and high drug accumulation in the tumor region owing to the enhanced permeability and retention (EPR) effect. This system allows for gradual or temporary release of free active drugs from the nanocarriers in a controlled fashion and a constant drug concentration in the tumor tissue [14–16]. It is well known that poly(l-Aspartic)-polymeric-drug conjugates have consistently been used for drug development as PAsp is biocompatible, biodegradable, and easily modified to be a drug conjugate [17, 18]. Similarly, the carboxyl group on the structure of Aspartic acid can be esterified with the hydroxyl group on the CA4 structure to form a nano-prodrug. As a result, the nano-drug delivery system constructed by chemical bonding is more stable than physical enwrapping and may be sensitive to tumor acidic environment [19].

Immune checkpoint blockade (ICB) that aims to reverse signals from the immunosuppressive TME is being driven as a primary treatment modality [20–22]. The efficacy of ICB-based cancer immunotherapy largely depends on the expression of PD-L1 in the tumor tissues and the recruitment of tumor-infiltrating lymphocytes (TILs) [23, 24]. Owing to the high interstitial pressure inside the tumor center, the infiltration of T cells is hard to get inside the central area of tumor. As a result, they are easier to exert anti-tumor effect in the periphery [25]. Numerous related reports also show that the overexpression of HIF-1α could induce the overexpression of PD-L1 in tumor tissues [26–29]. It is known that CA4 after delivered by nanoparticles in vivo could elicit central tumor necrosis and leaving normal tissues relatively impregnable, which exacerbate hypoxia in tumor tissue [30–33]. This phenomenon reveals that anti-PD-L1 should really be synergistic to inhibit tumor growth and reoccurrence in case of overexpression of PD-L1 during the treatment of CA4. Actually, as the landscape of anti-PD-1/PD-L1 clinical trials moves towards combination strategies, which may be more efficacious, a recent report revealed that 253 drug target groups (excluding PD-1/PD-L1) are being tested, which represents an increase of 129 new combined target groups in the past 3 years [34]. Hence, to design a combined treatment with aPD-L1 and CA4 for cancer is quite significant.

**Scheme 1:**
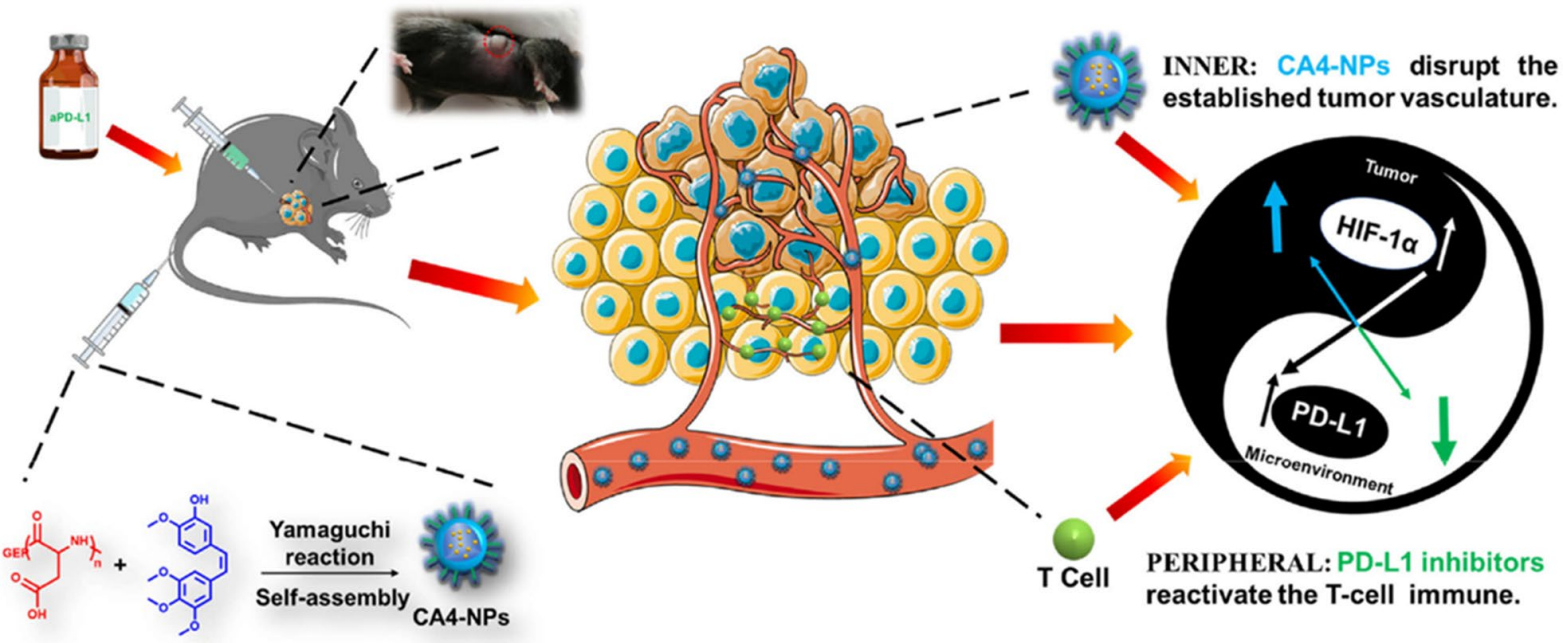
CA4-NPs arrest the tumor by disrupting the established central tumor vasculature, which can induce severe hypoxic conditions and high expression of HIF-1α in tumor tissues. The hypoxic condition can increase the expression of PD-L1 in tumor microenvironment, so the PD-L1 inhibitors can release the ‘immune brake’ to reactivate the T-cell immune response to the tumor effect in the peripheral area

Therefore, in this study, nanoparticles of poly(l-Aspartic acid)-poly(ethylene glycol)/combretastatin A4 (CA4-NPs) were prepared to improve the water solubility and prolong the half-life of CA4. CA4 itself has good selectivity for central tumor blood vessels and after being linked with PEG-b-PAsp by ester bond, CA4-NPs could offer significant advantages of EPR effect and acid-sensitive (pH = 5.5) effect to TME. And a combination of CA4-NPs plus aPD-L1 was tested in subcutaneous Hepa1-6 hepatic tumor models. The CA4-NPs effectively disrupted established tumor blood vessels, caused extensive tumor necrosis and inhibited tumor growth, but induced severe hypoxic conditions which resulted in the overexpression of PD-L1 in tumor tissues. Then co-administration of aPD-L1 can efficiently up-regulate the growth and proliferation of T cells and activate their attack and killing functions. As a result, this combination of treatment could achieve remarkable efficacy for HCC due to the regulation of tumor microenvironment (Scheme 1).

## Results and discussion

### Preparation and characterization of PEG-b-PAsp-g-CA4

**Fig. 1:**
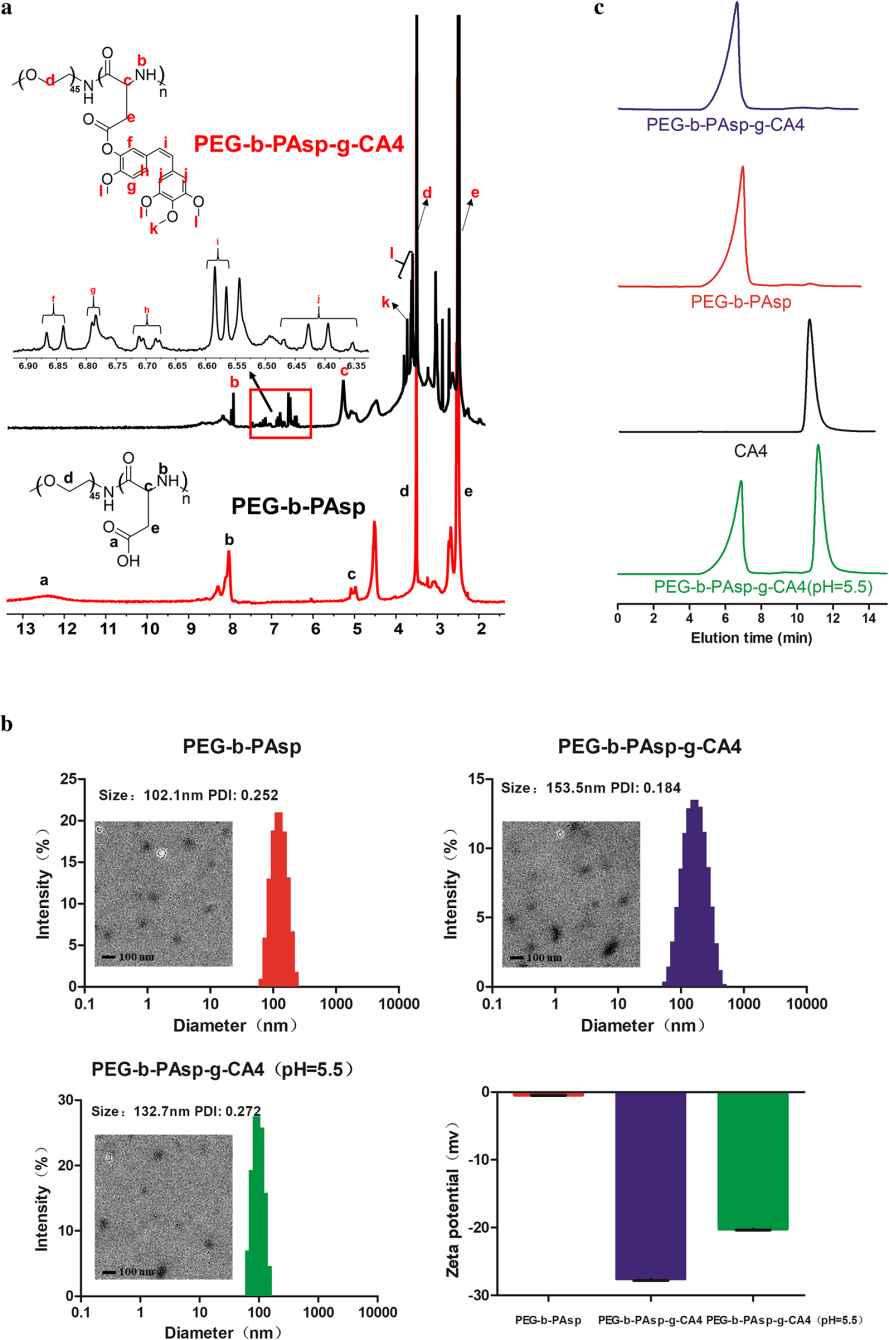

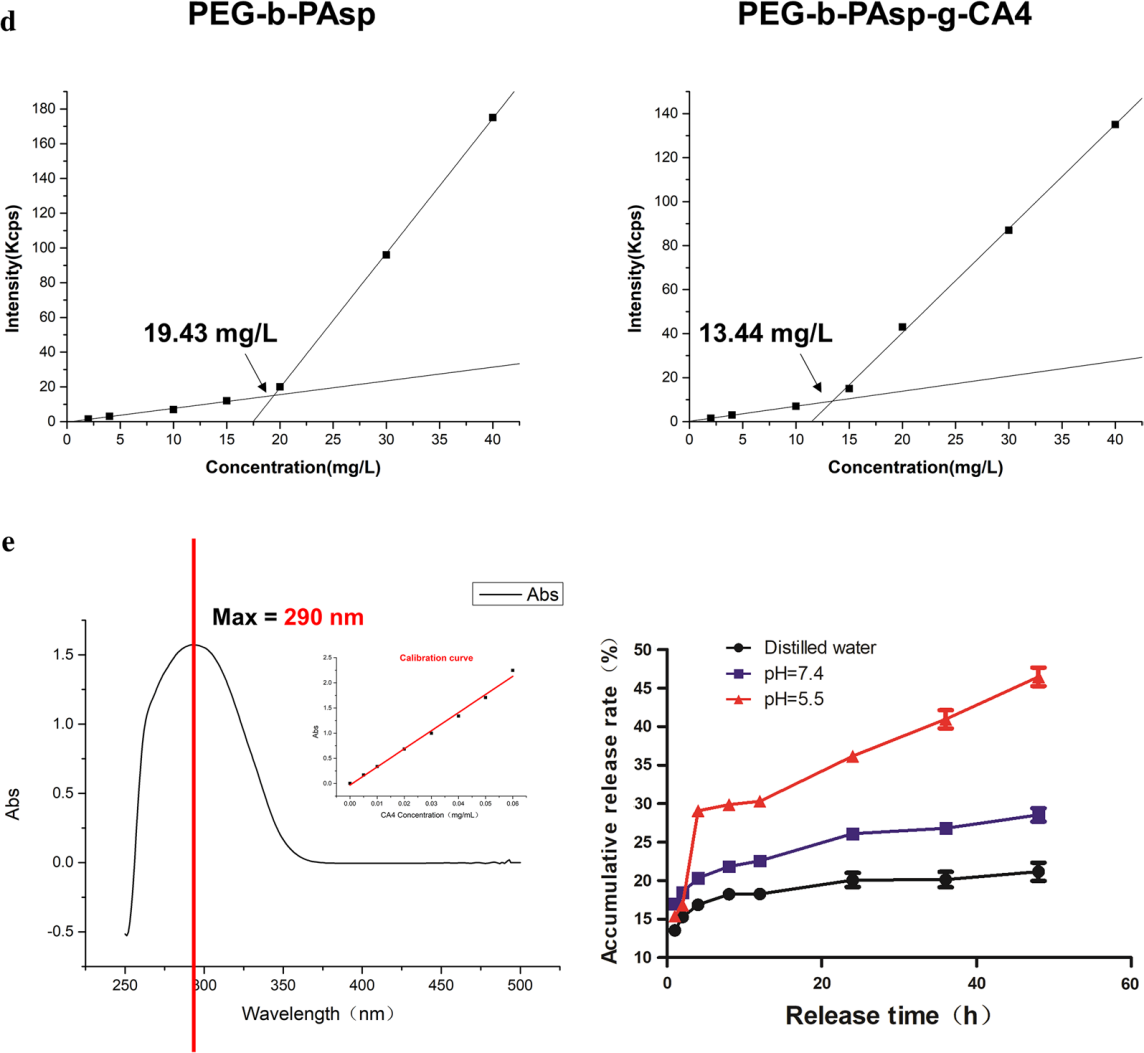
**a** ^1^H NMR spectra of PEG-b-PAsp and PEG-b-PAsp-g-CA4; **b** Representative TEM images, size distribution, and zeta potential of the PEG-b-PAsp and PEG-b-PAsp-g-CA4 (also including in pH = 5.5); **c** GPC curves of PEG-b-PAsp-g-CA4, PEG-b-PAsp, CA4, and PEG-b-PAsp-g-CA4 (in pH = 5.5); **d** Critical micellar concentration measurement of PEG-b-PAsp and PEG-b-PAsp-g-CA4 in water; **e** UV–vis absorption spectra of CA4 and drug release of PEG-b-PAsp-g-CA4 in pH 5.5/ 7.4 PBS buffer, distilled water

In this study, CA4 was conjugated to PEG-b-PAsp using a one-step Yamaguchi reaction catalyzed by 2,4,6-trichlorobenzoyl chloride and DMAP [14]. The chemical structure of the formed PEG-b-PAsp and PEG-b-PAsp-g-CA4 was confirmed by ^1^H NMR (Fig. 1a). The peaks around 3.51 (d) ppm were attributable to the protons (–CH_2_CH_2_O) in the PEG chain. The peak around 12.5 (a), 8.06 (b), 4.9 (c), and 2.7 (e) ppm are attributable to the protons (–COOH, –CONH–, –COCHNH–, CH_2_COOH) in the PAsp chain. Resonance peaks at 6.85 (f), 6.80 (g), 6.70 (h), 6.58 (i) and 6.40 (j) 3.83 (k) 3.71 (l) ppm were attributed to the presence of CA4. Since the particle size, ζ potential, and stability were considered as significant factors that determine the fate of nanodrugs, the appearance and stability of PEG-b-PAsp and PEG-b-PAsp-g-CA4 were characterized. The size of PEG-b-PAsp was 102.1 nm (PDI = 0.252) and after being linked with CA4, the size changed to 153.5 nm (PDI = 0.184), which was consistent with the result of TEM respectively (Fig. 1b). Notably, the diameters of these NPs remained less than 200 nm after drug loading, which was highly suitable for passive tumor targeting for drug delivery by the EPR effect and not easy to be cleared due to too smaller particle size. The zeta potentials of the NPs, which were − 0.44 (PEG-b-PAsp) and − 27.56 mV (PEG-b-PAsp-g-CA4), were suitable for intravenous administration. The result of the relative molecular mass of polymer was determined by Gel Permeation Chromatography (GPC) in Fig. 1c. The peak time of PEG-b-PAsp-g-CA4 was earlier than PEG-b-PAsp, which meant that relative molecular mass of CA4-NPs was bigger and no detectable free CA4 was found in the conjugates. The CMC value for PEG-b-PAsp and PEG-b-PAsp-g-CA4 was measured to be 19.43 mg L^−1^ and 13.44 mg L^−1^ respectively (Fig. 1d).

### In vitro drug release

The CA4-NPs in pH 5.5 PBS buffer after 48 h were also detected by DLS, TEM, and GPC to mimic the drug release action in acidic tumor microenvironment. The diameter was about 132.7 nm (PDI = 0.272) and zeta potentials were − 0.2 mv, which changed obviously after drug releasing (Fig. 1b). While compared with other groups in Fig. 1c, it can be vividly observed that the second peak (CA4) appeared after the ester bond was broken and CA4 released from nanoparticles in pH 5.5, which all proved that PEG-b-PAsp-g-CA4 had a better release in the TME directly. The final PEG-b-PAsp-g-CA4 conjugates had a CA4 drug loading content (DLC%) of 29.8 wt% as confirmed by UV–Vis spectrometry. The quantity of CA4 released in pH 7.4, pH 5.0 PBS buffer or distilled water was quantified by HPLC using a UV–Vis detector, as shown in Fig. 1e. CA4 releasing from CA4-NPs was 47.2 ± 2.3% at 48 h in pH 5.5 PBS buffer, greater than in pH 7.4 PBS buffer (27.3 ± 1.3%), and in distilled water (16.2 ± 2.1%). The CA4 releasing at lower pH was quick in 4 h and then became slow in next 44 h in pH 5.5, which revealed that the ester bond of was acid-sensitive to the TME and PEG-b-PAsp-g-CA4 can reach the concentration of the drug quickly and last long next.

### In vitro cytotoxicity and uptake of the CA4-NPs experimental section

**Fig. 2:**
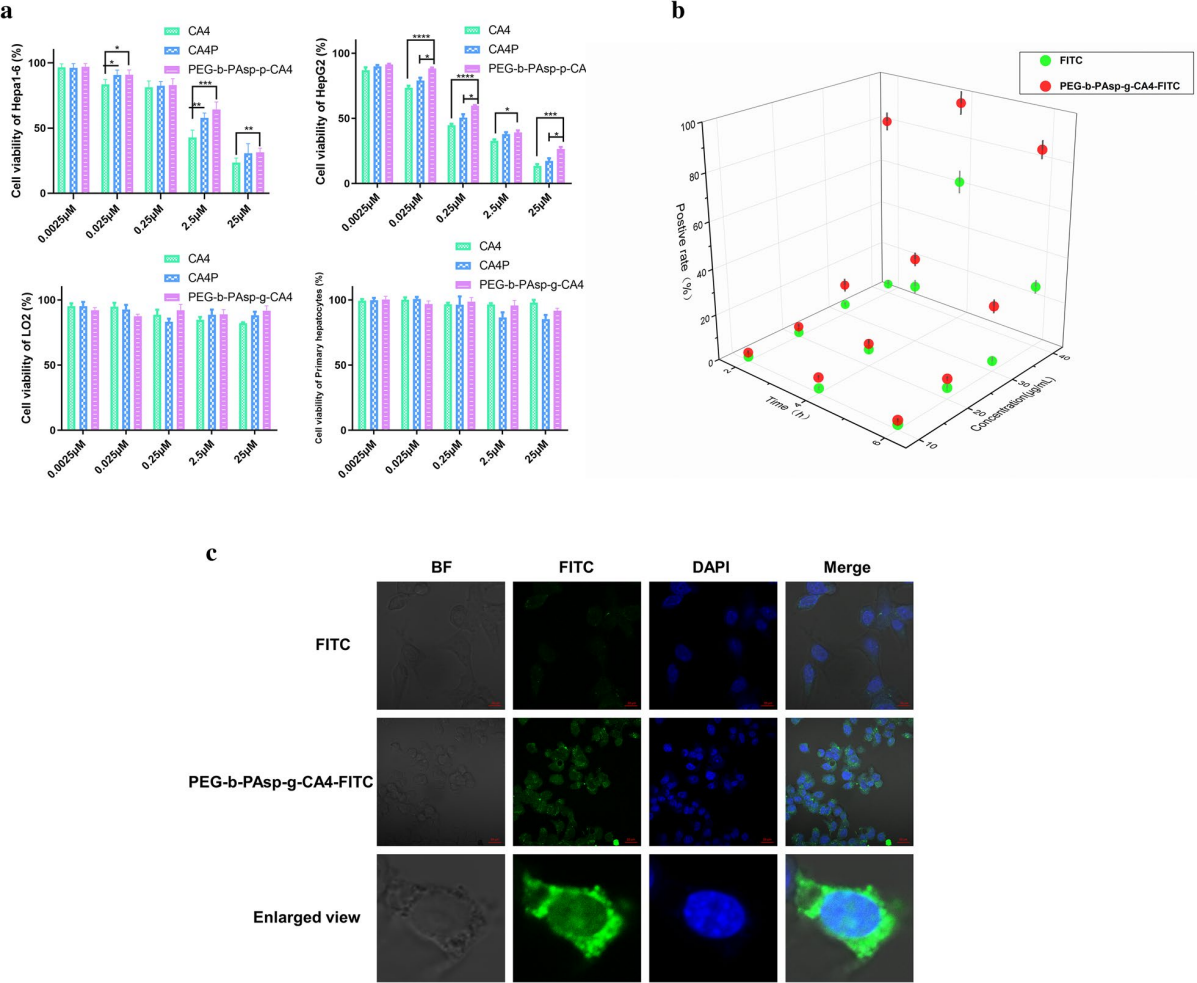
The cytotoxicity and uptake of the CA4-NPs. **a** Cytotoxicity of CA4, CA4P, PEG-b-PAsp-g-CA4 to Hepa1-6, HepG2, primary hepatocytes, LO2; **b** Fluorescence intensity of positive Hepa1-6 cells mixed with FITC and PEG-b-PAsp-g-CA4-FITC under different condition (Concentration of FITC and PEG-b-PAsp-g-CA4-FITC (on the FITC basis) varied from 10 to 40 μg mL^−1^ and incubated time was 2, 4, 6 h.). Cellular uptake was analyzed by fluorescence-activated cell sorting (FACS); **c** Confocal images of Hepa1-6 cells after a 4 h incubation with 40 μg FITC mL^−1^ and PEG-b-PAsp-g-CA4-FITC (on the FITC basis). Scale bar: 20 µm. Significance between each pair of groups was calculated using Student’s t-test. *p < 0.05, **p < 0.01, ***p < 0.001, ****p < 0.0001. The values are presented as the mean ± SD, n = 6

Cytotoxicity of the different drugs was respectively evaluated by MTT assay against Hepa1-6, HepG2, LO2 cells, and primary hepatocytes were used in this study. As shown in Fig. 2a, 0.0025–25 μM of CA4, CA4P, CA4 -NPs exhibited cytotoxicity to Hepa1-6 and HepG2 cells. Not like small molecules, the release of drug from nanoparticles was sustained and controlled. As a result, the cytotoxicity of CA4 -NPs was lower than CA4 in limited time because the drugs linked with nanoparticles have not been completely released to exert their effects on cells. Meanwhile, the prepared CA4-NPs showed no cytotoxicity to LO2 cells and primary hepatocytes, which meant that PEG-b-PAsp can be used as a safe drug carrier.

To visualize the intracellular behavior of CA4 -NPs, we labeled the nanomaterials by fluorescein isothiocyanate (FITC) and investigated their uptake by Hepa 1–6 cells using confocal laser scanning microscopy (CLSM) and fluorescence-activated cell sorting (FACS). The concentration of FITC and PEG-b-PAsp-g-CA4-FITC (on the FITC basis) varied from 10 to 40 μg mL^−1^ and the incubated time with Hepa1-6 cells was 2, 4, 6 h. The highest cell positive rate was over 90% detected by flow cytometry 4 h later and at a concentration of 40 μg mL^−1^ of CA4 -NPs. Nonetheless, a lower cell positive rate was obtained after being incubated after 6 h than it after 4 h, which may result from the enhanced cytotoxicity of FITC after being incubated longer time (Fig. 2b). And dependent on this condition, cellular localization experiments were performed using CLSM. Hepa1-6 cells treated with PEG-b-PAsp-g-CA4-FITC (40 μg mL^−1^) presented significantly higher fluorescent signal after 4 h in Fig. 2c, indicating a more cellular uptake than that of free FITC in the nuclei.

### Pharmacokinetics and biodistribution of CA4-NPs

**Fig. 3:**
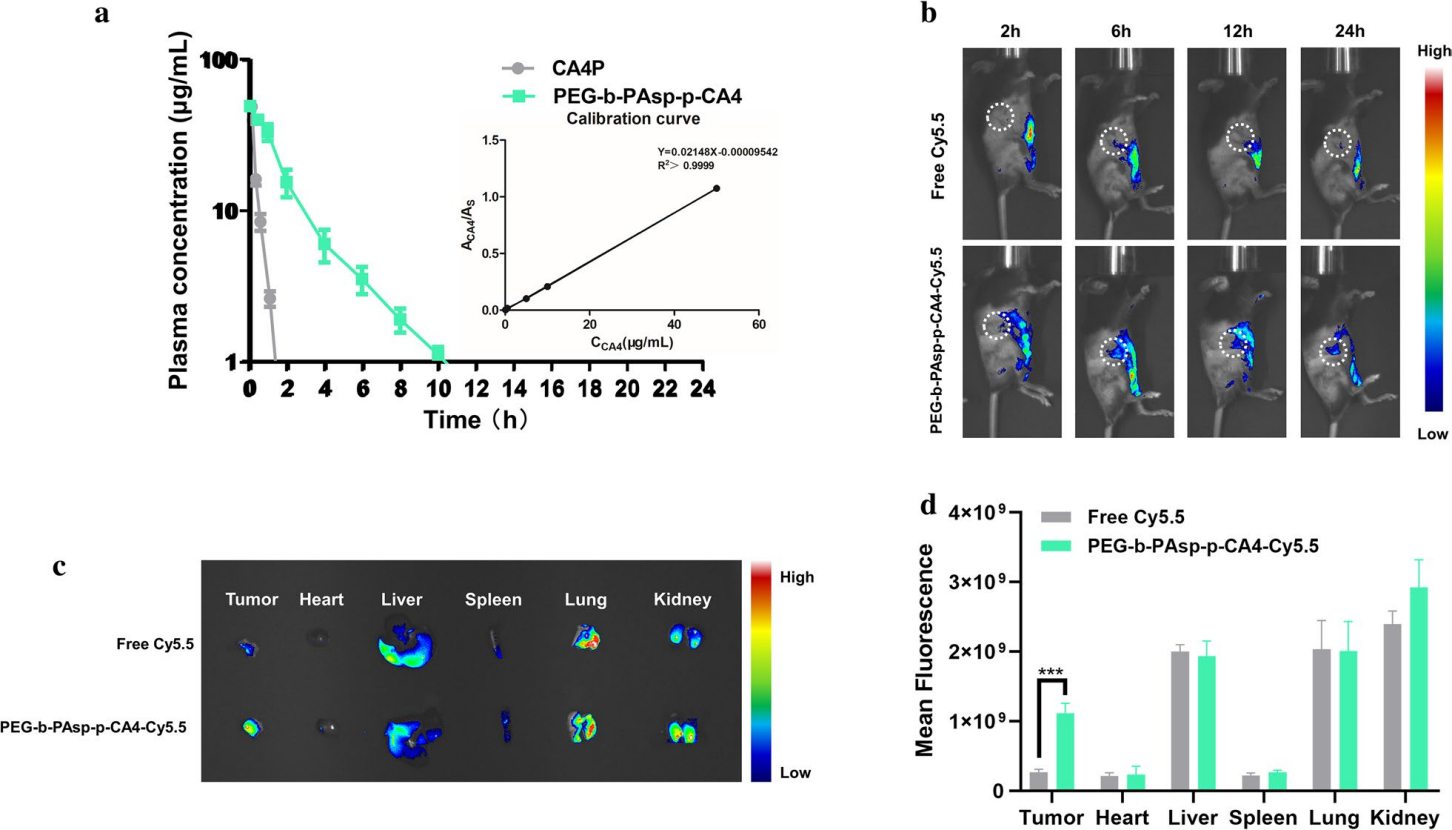
Pharmacokinetics and biodistribution of CA4-NPs. **a** Pharmacokinetics of CA4P and PEG-b-PAsp-g-CA4; **b** In vivo fluorescence images of Hepa1-6 tumor-bearing mice taken at different time points intravenously administrated with Cy5.5 and PEG-b-PAsp-g-CA4-Cy5.5; **c** Ex vivo fluorescence images of the major organs (heart, liver, spleen, lungs, and kidneys) and tumors at 24 h intravenous administration; **d** Quantitative analysis of ex vivo Cy5.5 fluorescence intensity of the organs and tumor. Significance between each pair of groups was calculated using Student’s t-test. *p < 0.05, **p < 0.01, ***p < 0.001, ****p < 0.0001. The values are presented as the mean ± SD, n = 3

To compare the circulation time of CA4 -NPs, and CA4P in vivo, SD rats were administered with CA4-NPs or CA4P at a dose of 50.0 mg kg^−1^ based on CA4. As shown in Fig. 3a, the plasma concentration–time profiles fitted in Non-compartment models well, and the main pharmacokinetic parameters of PEG-b-PAsp-g-CA4 were as follows: C_max_ 50.96 ± 5.41 μg mL^−1^, T_1/2_ 2.18 ± 0.22 h, MRT 2.26 ± 0.17 h, AUC_0−τ_ 120.66 ± 10.37 mg h^−1^ L^−1^, AUC_0−∞_ 123.21 ± 10.85 mg h^−1^ L^−1^. In contrast, for free CA4P, C_max_ 49.18 ± 1.02 μg mL^−1^, T_1/2_ 0.24 ± 0.02 h, MRT 0.28 ± 0.02 h, AUC_0−τ_ 15.32 ± 1.49 mg h^−1^ L^−1^, AUC_0−∞_ 15.51 ± 1.56 mg h^−1^ L^−1^. The pharmacokinetic data indicated that PEG-b-PAsp-g-CA4 has a longer blood circulation time than CA4P, achieving more tumor accumulation and retention to destroy tumor blood vessels for a longer period, which can provide higher anti-tumor efficacy by administering lower doses at longer intervals. Pharmacokinetic data was analyzed using WinNonLin software version 7.0.

To verify the behaviors in vivo, free Cyanine5.5 NHS ester (Cy5.5, SE) and PEG-b-PAsp-g-CA4-Cy5.5 were monitored by in vivo imaging method. As shown in Fig. 3b, free Cy5.5 had no significant tumor accumulation at each predetermined time. Notably, the fluorescence of Cy5.5 delivered by PEG-b-PAsp-g-CA4 peaked at around 6 h. The fluorescence intensity of PEG-b-PAsp-g-CA4-Cy5.5 remained about 4 times higher in tumors ex vivo that of free Cy5.5 at 24 h after administration (p < 0.001, Fig. 3c, d). This phenomenon can be explained by the EPR effect and acid-sensitive (pH = 5.5) effect of CA4 NPs to TME, and the excellent selectivity of CA4 for tumor central blood vessels, which is conducive for CA4-NPs to exert a better therapeutic effect compared with the CA4 treatment group (Additional file 1: Figure S1). At the stage of drug accumulation in vivo, free molecule showed a little bit faster elimination for overall observation in the major organs (heart, liver, spleen, lungs, and kidneys) when compared to the NPs group in Fig. 3d and Additional file 1: Figure S2. In summary, the CA4-NPs was able to efficiently accumulate and retain in tumors after intravenous injection, which could be beneficial for anticancer applications in vivo.

### Mechanism of combination aPD-L1 with CA4-NPs

**Fig. 4:**
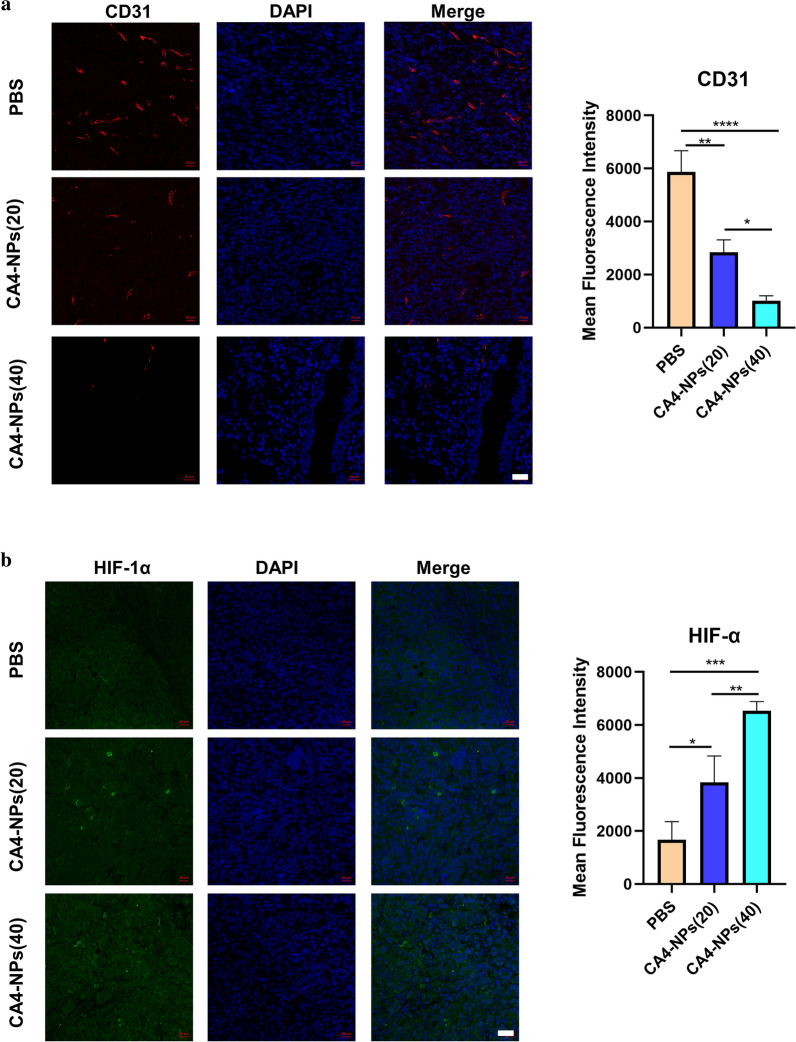

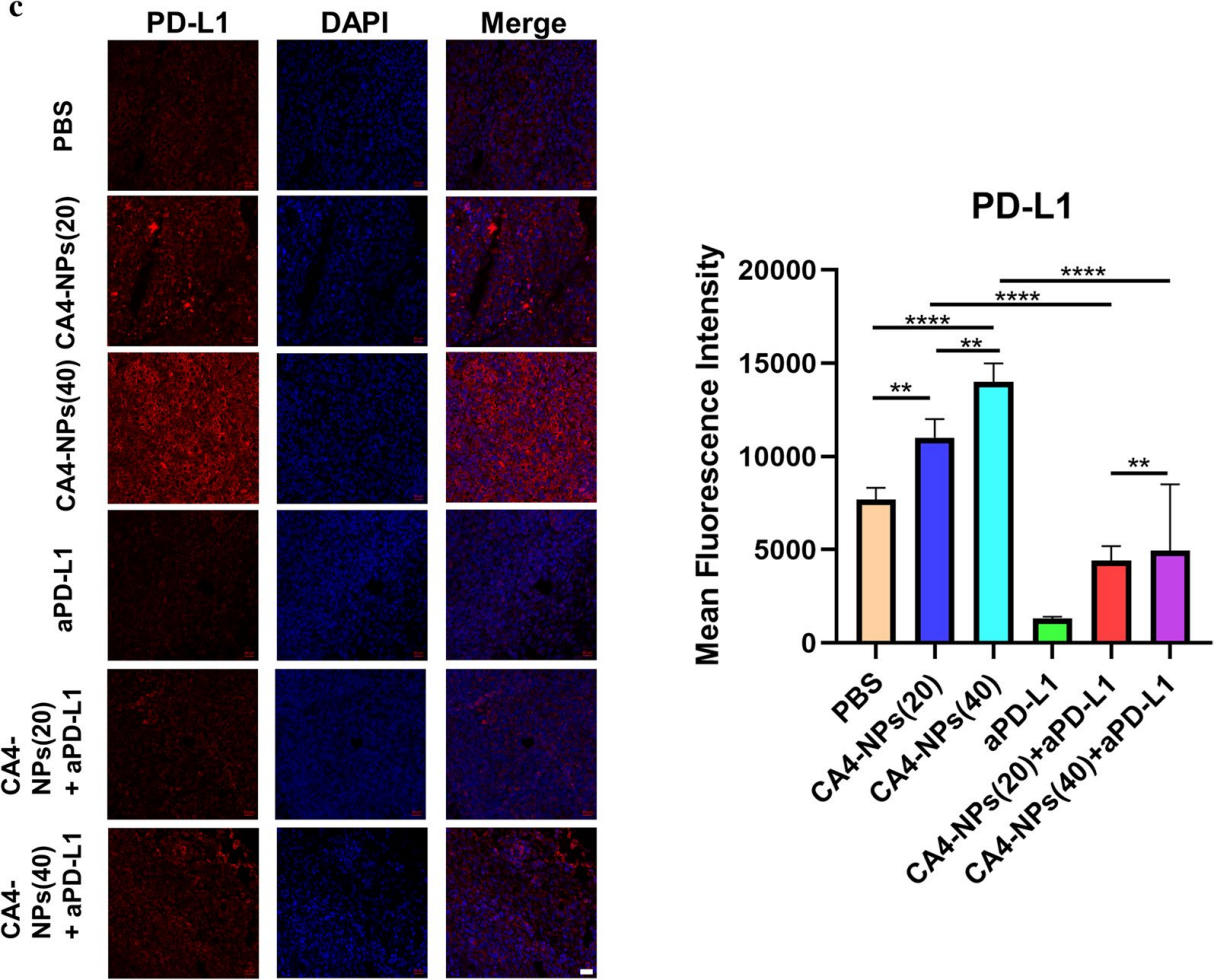
Mechanism of combination aPD-L1 with CA4-NPs. Immunofluorescence staining for **a** CD31, **b** HIF-1α, **c** PD-L1 expression of tumor tissues 48 h post-treatment and quantitative immunofluorescence analysis. CA4-NPs (20) represented that the dose of CA4-NPs was 20 mg kg^−1^ and CA4-NPs (40) represented that the dose of CA4-NPs was 40 mg kg^−1^. Scale bar: 20 μm. Significance between each pair of groups was calculated using Student’s t-test. *p < 0.05, **p < 0.01, ***p < 0.001, ****p < 0.0001. The values are presented as the mean ± SD, n = 3

In order to investigate the vascular state after 48 h post-injection of CA4-NPs, we used immunofluorescence technology to stain CD31 of tumor tissues, which indicated that the vascular density in the CA4-NPs (40) group was lower than that in the CA4-NPs (20) group, and the vascular density in the CA4-NPs (20) group was lower than that in the PBS group (Fig. 4a). These results showed that CA4-NPs performed good triple target effects—EPR effect, acid-sensitive (pH = 5.5) effect to TME, and high selectivity for tumor blood vessel, and strong dose-dependent vascular disruption in tumor tissues**.** Considering that CA4-NPs treatment could induce a severe hypoxic microenvironment in solid tumors because of vascular disruption, we then compared the level of HIF-1α in Hepa1-6 tumor tissues under immunofluorescence staining. As a result, CA4-NPs treatment increased the expression of HIF-1α in tumor tissues significantly. The expression of HIF-1α in the CA4-NPs (20) group was higher than that in the PBS group, and the level of HIF-1α in the CA4-NPs (40) group was the highest among the three groups after 48 h intravenous injection (Fig. 4b). These results revealed that CA4-NPs treatment could block the vessels and induce severe hypoxic conditions that HIF-1α expressed higher in tumor tissues. Thus, we continued to compare the level of PD-L1 in Hepa1-6 tumor tissues dependent on the results of HIF-1α, and a similar conclusion was obtained that CA4-NPs treatment also increased the expression of PD-L1 in tumor tissues significantly. Nonetheless, the level of PD-L1 in the combined treatment (CA4-NPs with aPD-L1) significantly descended compared to the single administration group (CA4-NPs) after 48 h intravenous injection, and single administration group (aPD-L1) showed the most obviously inhibitory effect on PD-L1 expression level among the six groups (Fig. 4c), which revealed that CA4-NPs together with aPD-L1 might produce a better treatment effect compared to the single administration.

### In vivo anti-tumor efficacy

It is not ideal for tumor treatment when the overexpression of PD-L1 in tumor tissues induced by hypoxic microenvironment after the use of CA4-NPs, which is quite possible to result in reoccurrence of tumor cells. To address this serious problem, we combined aPD-L1 with CA4-NPs simultaneously dependent on the results of the pharmacokinetics and biodistribution of CA4-NPs and exploration of mechanism of co-administration with aPD-L1 before. A subcutaneous Hepa1-6 hepatocellular carcinoma mouse model was established on female C57BL/6 mice as described above.

**Fig. 5:**
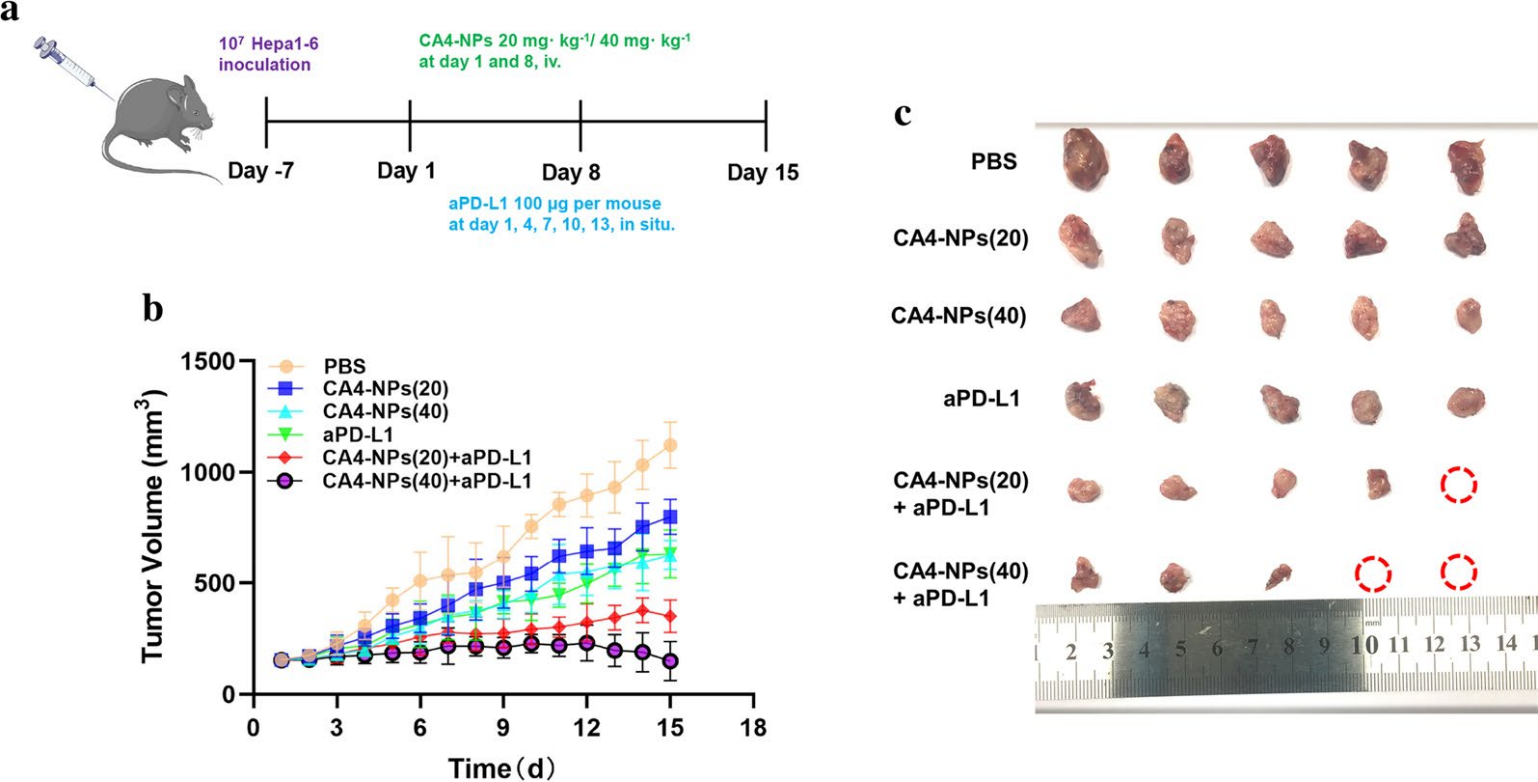

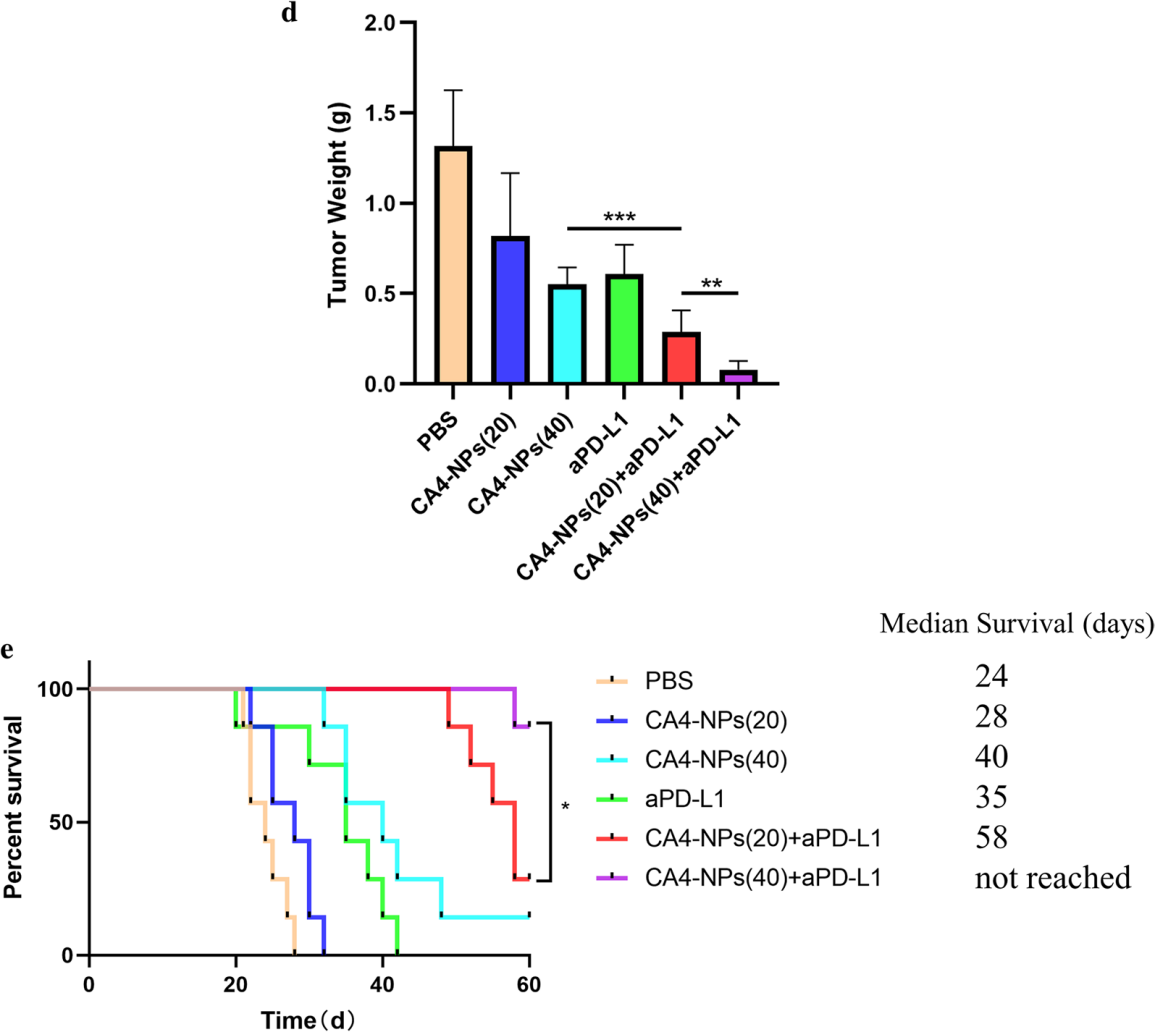
Co-administration of anti-tumor efficacy In vivo. **a** Treatment scheme of subcutaneous Hepa1-6 tumors with CA4-NPs and aPD-L1; **b** Tumor volume growth curves following different treatments; **c** Representative images of the excised tumors after different treatments; shown are PBS-treated mice and mice receiving after injection with CA4-NPs (20 mg kg^−1^), CA4-NPs (40 mg kg^−1^), aPD-L1, CA4-NPs (20 mg kg^−1^) + aPD-L1, CA4-NPs (40 mg kg^−1^) + aPD-L1; **d** Tumor weights from the different groups on day 15; **e** The survival percentages of the tumor-bearing C57BL/6 mice (n = 7 biologically independent samples). Significance between each pair of groups was calculated using Student’s t-test. *p < 0.05, **p < 0.01, ***p < 0.001, ****p < 0.0001. The values are presented as the mean ± SD, n = 10

When the tumor volume reached about 150 mm^3^, the mice were randomly divided into 6 groups: PBS**,** CA4-NPs (20 mg kg^−1^), CA4-NPs (40 mg kg^−1^), aPD-L1, CA4-NPs (20 mg kg^−1^) + aPD-L1, CA4-NPs (40 mg kg^−1^) + aPD-L1 (20 mg kg^−1^). CA4-NPs (20, 40 mg kg^−1^) and PBS were separately administrated to mice via intravenous injection at day 1, 8 and aPD-L1 was administrated to mice via intratumoral injection at day 1, 4, 7, 10, 13, and the tumor volumes were continuously monitored for 15 days (Fig. 5a). The dosage of aPD-L1 was 50 µL for each mouse. Tumor growth curves were shown in Fig. 5b where fast-growing tumor volumes were observed in PBS and CA4-NPs (20) treated mice. In contrast, a modest inhibition of tumor growth was achieved in mice receiving CA4-NPs (40) and aPD-L1, and also as shown in Fig. 5c, d, a 58.1% or 53.7% reduction of the relative tumor weight at 15th day was observed respectively. Notably, the most effective anti-tumor effect was realized in CA4-NPs (20,40) with aPD-L1 treated group that tumor weight at the last day was reduced by 78.2% and 89.7%, which may be benefited from the combined blocking effect of aPD-L1 in TME.

As depicted in Fig. 5e, the median survival time of mice receiving CA4-NPs (20) and CA4-NPs (40) were 28 and 40 days, respectively. The combination treatment significantly prolonged survival of Hepa1-6 tumor-bearing mice compared with the single drug treatment groups. The median survival time of mice in the combination groups was more than 58 days. The above observations unambiguously evidenced the remarkable anticancer efficacy of the combinenation strategy.

**Fig. 6:**
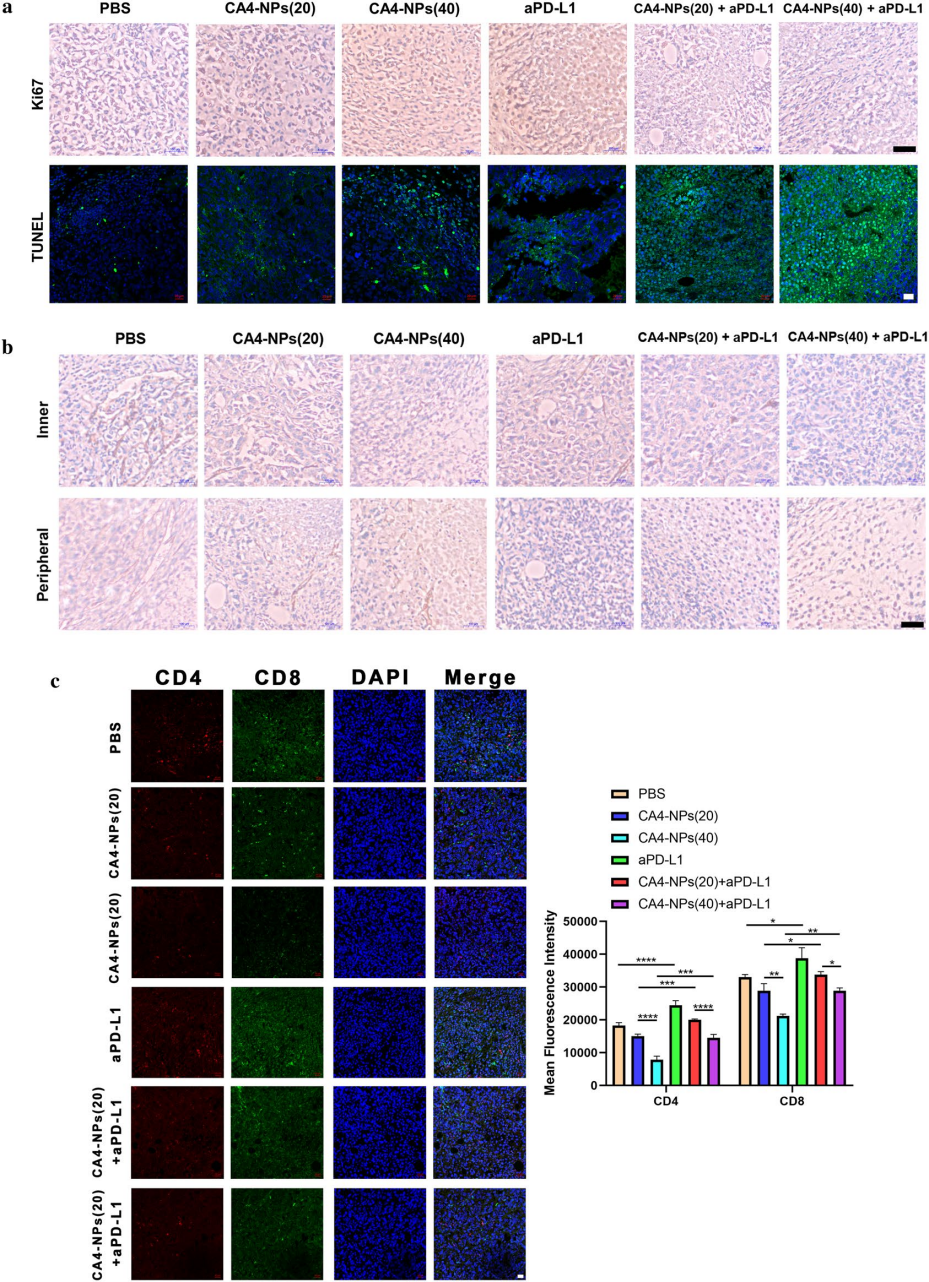

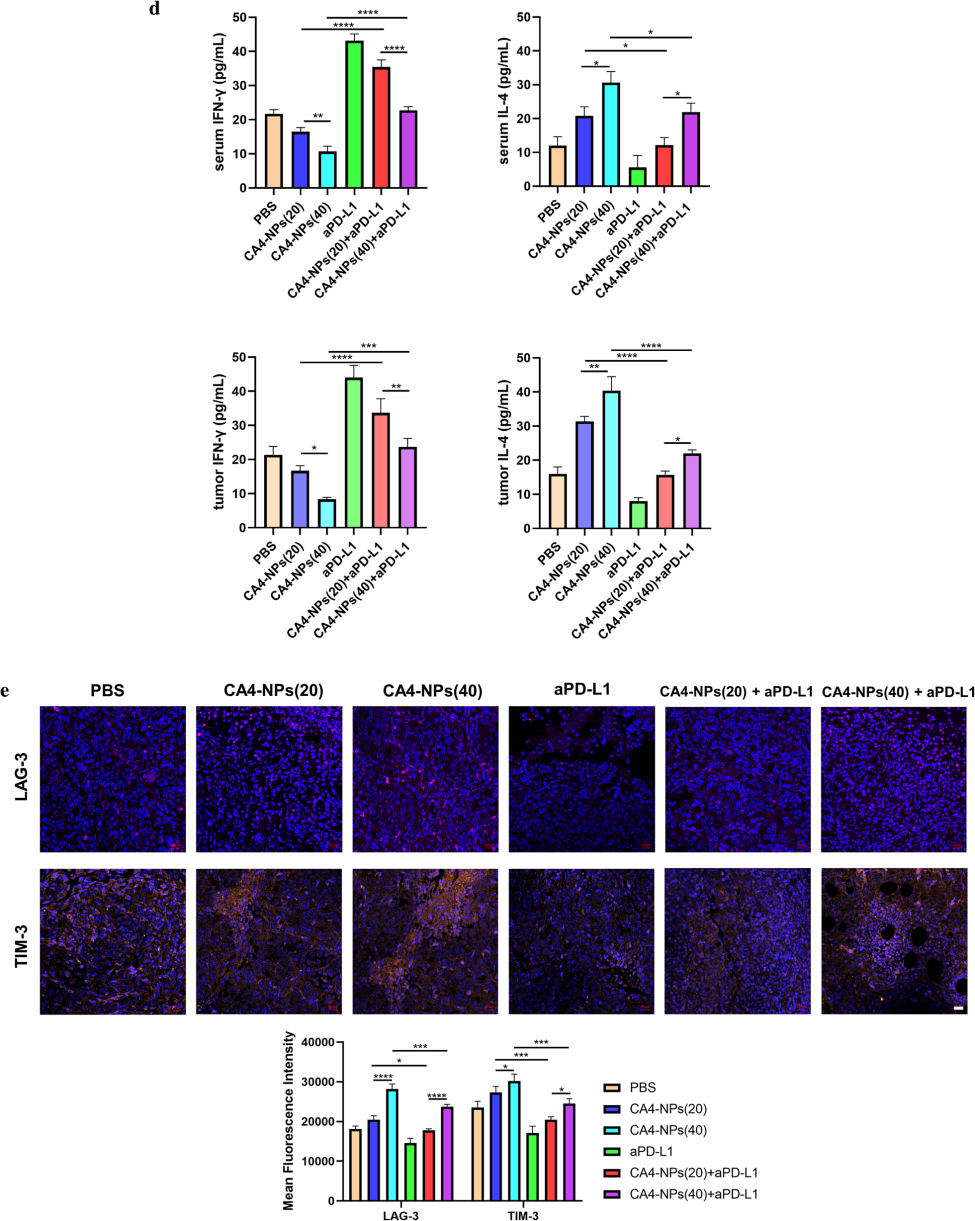
**a** Ki67 and TUNEL staining of tumor tissues after different treatments; **b** Immunohistochemical reactivity for blood vessel endothelial cell marker CD31 in subcutaneous Hepa1-6 bearing mice;** c** Double-color immunofluorescence staining for CD4 (Red) and CD8(Green) expression of tumor tissues, and quantitative immunofluorescence analysis; **d** The secretions of cytokines in serum and tumor site were measured by ELISA (levels of IFN-γ and IL-4); **e** Immunofluorescence staining for LAG-3 and TIM-3 expression of tumor tissues, and quantitative immunofluorescence analysis. Black scale bar = 100 μm and white scale bar = 20 μm. Significance between each pair of groups was calculated using Student’s t-test. *p < 0.05, **p < 0.01, ***p < 0.001, ****p < 0.0001. The values are presented as the mean ± SD, n = 3

**Fig. 7:**
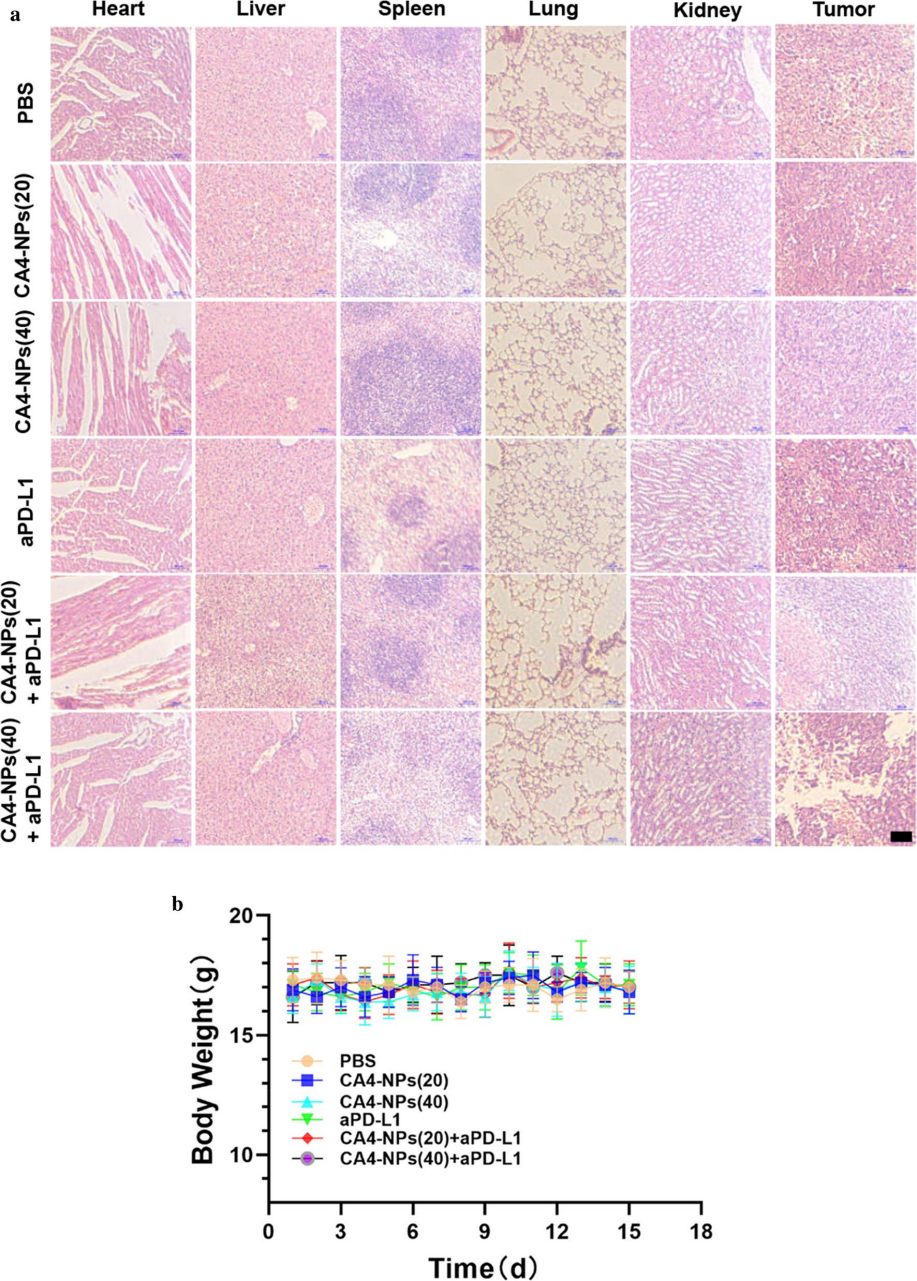
**a** Hematoxylin & eosin (HE)-staining images of heart tissues, liver tissues, spleen tissues, lung tissues, kidney tissues, and tumor tissues under light microscopy; **b** Mice weights after different treatments. N = 10, scale bars: 100 μm

Additionally, to investigate the anti-tumor therapeutic mechanism of the combined treatment, histological staining analyses were applied to the isolated tumors, including Ki67, TUNEL and hematoxylin and eosin (H&E). Less Ki67-positive cells (proliferative cells) and more TUNEL-positive cells (apoptotic cells) could be found in the co-administration treated tumors (Fig. 6a). On the contrary, more proliferative tumor cells and fewer apoptotic tumor cells were observed in the case of PBS and single administration treated mice. We next analyzed CD31 expression in tumors for immunohistochemistry (IHC) to investigate tumor microvascular changes after different treated groups. The CD31 staining images of mice in CA4-NPs group were taken in the peripheral area of the tumor (Fig. 6b). CA4-NPs significantly disrupted the established tumor vascular and suppressed tumor growth (inner area), while continuous administration groups of aPD-L1 showed obviously inhibitory revascularization and regrowth of the treated tumor (peripheral area), which may be contributed to the treatment of tumor reoccurrence. Also, the number of CD4^+^ T cells and CD8^+^ T cells were significantly increased in combination groups when compared to single groups (Fig. 6c). Previous research suggested that IFN-γ and IL-4 are representative of Th1 and Th2 cytokines [35]. The production of signature Th1 cytokine IFN-γ increased more both in serum and tumor site by combinated strategy, whereas for the Th2 cytokine IL-4, it decreased significantly in combination groups in Fig. 6d. It is also widely reported that the expression of Tim-3 and Lag-3 on different tumor cells can be served as potential biomarkers to predict the response to anti-PD-1/PD-L1 therapy [36–38]. So we finally investigated the LAG-3 and TIM-3 expression in tumor tissues by immunofluorescence staining and it can obserbed that tumors treated by CA4-NPs expressed less LAG-3/TIM-3 after combinatd with aPD-L1 in Fig. 6e. All these mechanism results confirmed the successful anti-tumor therapy by co-administration. Furthermore, in Fig. 7, compact tumor tissue was found for PBS and CA4 treated mice, while a remarkable reduction of tumor cells and more vacancies was observed after co-administration. Meanwhile, no significant loss of body weight was observed in all groups, indicating the safety and biocompatibility of the nanoparticles used here, which was further confirmed by H&E staining of major organs.

## Conclusion

In this study, a combination of poly(L-Aspartic acid)-poly(ethylene glycol)/combretastatin A4 (CA4-NPs) nanoparticles with aPD-L1 was developed for systemic treatment of HCC. The use of CA4-NPs was aimed to cause extensive tumor necrosis mainly due to the triple target effects—EPR effect, acid-sensitive (pH = 5.5) effect to TME and good selectivity of CA4 for tumor blood vessel. Also, the circulation time of CA4-NPs in vivo was about 10 times that of CA4P. However, CA4-NPs also induced overexpression of PD-L1 that was confirmed by immunofluorescence staining in the treated tumors. Therefore, the aPD-L1 was applied to efficiently up-regulate the growth and proliferation of T cells and activate their attack and killing functions. The combination of PEG-b-PAsp-g-CA4 at 40 mg kg^−1^ with aPD-L1 (50 µL) showed a nearly 90% tumor suppression rate in a Hepa1-6 subcutaneous tumor model with low systemic toxicity. These findings indicated that the two-pronged attack of aPD-L1 with a strategy of VDAs, CA4-NPs, was really a potential therapeutic approach for HCC treatment.

## Methods

### Materials

All reagents and solvents were commercially available and used without additional treatment. l-Aspartic acid benzyl ester was purchased from Shanghai Bide Pharmatech Co., LTD. Triphosgene was purchased from Shanghai Aladdin Biotechnology Co., LTD. Combretastatin A4 (CA4) and combretastatin-A4 phosphate (CA4P) were purchased from Nanjing Chunqiu Biotechnology Co., LTD. aPD-L1 (BE0101) used in vivo purchased from Bio X Cell. Cyanine5.5 NHS ester (Cy5.5, SE) was purchased from Beijing Fanbo Biochemicals Co., LTD. Dulbecco’s modified Eagle’s medium (DMEM), fetal bovine serum (FBS), 3-(4,5-dimethylthiazol-2-yl)-2,5-diphenyltetrazolium bromide (MTT), trypsin–EDTA, penicillin–streptomycin, dimethyl sulfoxide (DMSO), and 4,6-diamidino-2-phenylindole (DAPI) were obtained from Gibco. 96 well plates, 6-well plates, and 10 mL graduated sterile centrifuge tubes were purchased from Jiangsu KeyGen Biotechnology Co., LTD. Other reagents and chemicals were at least analytical reagent grade.

### Preparation of PEG-b-PAsp-g-CA4

**Fig. 8:**
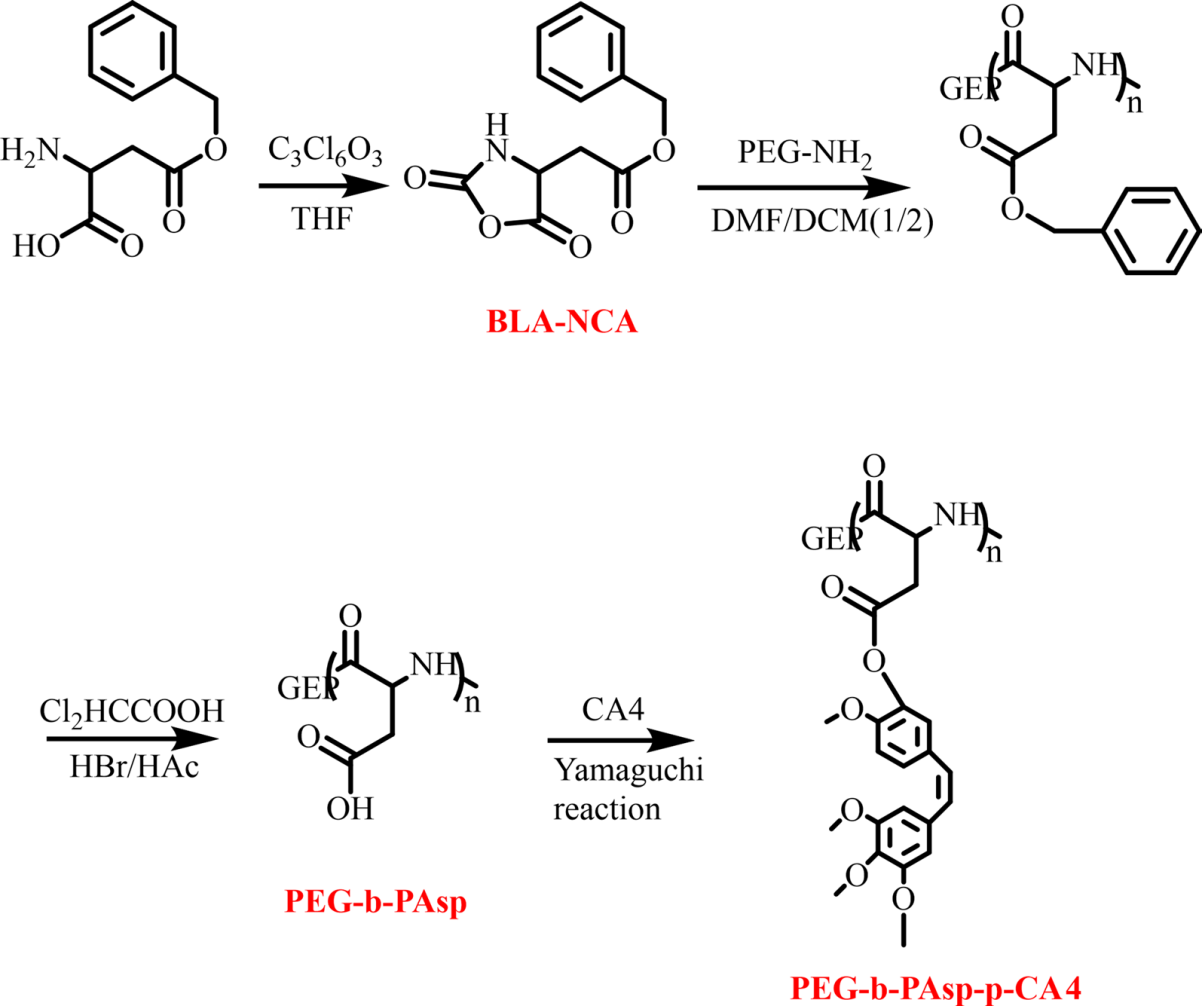
Synthesis procedures of PEG-b-PAsp-g-CA4

l-Aspartic acid benzyl ester and triphosgene (C_3_Cl_6_O_3_) were added into anhydrous THF respectively, then were mixed, stirred, and heated for 2 h at 55 °C. *N*-carboxy-a-amino acid anhydrides of b-Benzyl-l-aspartate (BLA-NCA) was obtained after being added n-hexane for crystallization and suction. The diblock copolymer (PEG-b-PAsp) was firstly synthesized via the amine-initiated ring-opening polymerization (ROP) of BLA-NCA. Briefly, BLA-NCA was dissolved completely in DMF/DCM followed by the addition of initiator which had been dissolved in DCM. Then, the reaction mixture was stirred for 5 days at 35 °C under a dry nitrogen atmosphere and the crude products were precipitated in tenfold excess of cold diethyl ether and isolated by centrifugation. After washed twice with diethyl ether, the products were dried in a vacuum. Block copolymers of PEG-b-PAsp were obtained by deprotection of PEG-PBLA in HCCl_2_COOH/HBr/HAc solution. After stirred for 3 h under an ice bath, the solution was precipitated in a large amount of cold diethyl ether and isolated by centrifugation. The solid was dissolved in DMSO. The solution was dialyzed against distilled water (MWCO 3500) and then solid PEG-b-PAsp was obtained by lyophilization. CA4 was grafted to PEG-b-PAsp by the Yamaguchi reaction. Briefly, PEG-b-PAsp was dissolved in anhydrous *N*, *N*-dimethylformamide (DMF) in a glass reactor, then CA4, 2,4,6-trichlorobenzoyl chloride, DMAP and triethylamine were dissolved in anhydrous DMF and added to the above mixture. The reaction was allowed to proceed at room temperature for 2 h. After that, the reaction mixture was precipitated into excess diethyl ether, re-dissolved in DMF, and dialyzed against distilled water (MWCO 3500). The final product poly(l-aspartic acid)-graft-methoxy poly(ethylene glycol)/combretastatin A4 (PEG-b-PAsp-g-CA4) was obtained after lyophilization (Fig. 8).

### Characterization

^1^H NMR spectra of the polymers were recorded on a Bruker 400 MHz nuclear magnetic resonance instrument using DMSO as the solvents. Gel permeation chromatography (GPC) was used to analyze the molecular weights and molecular weight distributions (Mw / Mn) of the polymers. GPC of PEG-b-PAsp was measured at room temperature with a Waters 1525 chromatograph equipped with a Waters 2414 refractive index detector. H_2_O was used as eluents with a low rate of 1.0 mL/min and narrowly distributed polyethyleneglycol was used as standard. The size and surface charge of the nanocarrier was investigated on Malvern ZetasizerNano ZS 90 zeta potential analyzer. Ultraviolet–visible (UV–vis) spectra were collected using a LAMBDA-35 spectrometer. CA4 loading content was calculated by measuring the UV–Vis absorbance at 290 nm.

Transmission electron microscopy (TEM) was performed on a JEOL-2100 with an accelerating voltage of 200 kV. TEM samples were prepared by drop-casting dispersion onto copper grids covered by carbon film. Confocal images were acquired using a Zeiss confocal laser scanning unit mounted on an LSM 710 fixed-stage upright microscope (CLSM).

### In vitro release profiles

In vitro release of CA4 from PLG-CA4 was conducted using a dialysis method. Briefly, 3.0 mg PLG-CA4 was dissolved in 10 mL distilled water, PBS solution at pH 7.4 or 5.5, and bovine albumin (BSA), respectively. Samples were sealed in a dialysis bag (MWCO 3500 Da) and incubated in 45 mL of PBS at 37 °C with a shaking rate of 100 rpm. At predetermined time points (1, 2, 4, 8, 12, 24, 36, 48 h), 3 mL of the incubated solution was taken out and replaced with fresh media. The concentration of CA4 in the released media was determined by HPLC at 290 nm with a mobile phase of acetonitrile and water (80/20, v/v), as described above.

### Critical micelle concentration (CMC)

The CMC was determined using a Malvern Zetasizer, NANO ZS90 (Malvern Instruments Limited, U.K.) equipped with a laser light scattering spectrometer at 632.8 nm under room temperature (25 °C). Solutions containing different concentrations of PEG-b-PAsp and PEG-b-PAsp-g-CA4 were tested, and the light scattering intensity was recorded for each concentration analyzed.

### Cytotoxicity

The cytotoxicity was analyzed by the MTT method. Primary hepatocytes were isolated and cultured according to a previous method and with the modification of our team [39]. Hepa1-6 cells, HepG2 cells, and LO2 cells were cultured in DMEM medium with 10% FBS and primary hepatocytes were cultured in William's Medium E (supplemented with 5% fetal bovine serum, 100 U/ml penicillin, 100 μg ml^−1^ streptomycin, 1 × ITS and 5 nM dexamethasone) at 37 °C under 5% CO_2_. Briefly, Hepa1-6 cells, HepG2 cells, LO2 cells and primary hepatocytes were seeded in 96-well plates at a density of 1 × 10^5^ cell per well and cultured at 37 °C. CA4, CA4P and PEG-b-PAsp-g-CA4 with increasing w/w ratio were added, after incubation for 48 h, 20 μL MTT solution (5 mg mL^−1^) was added to each well. After incubation for 4 h, 100 μL dimethyl sulfoxide was added to replace the media to dissolve formazan, the absorbance was analyzed at a wavelength of 490 nm with a microplate reader.

### Cellular uptake and intracellular trafficking

Hepa1-6 cells were seeded into 24-well plates and then were incubated with the polyplexes prepared with FITC (10, 20, 30, and 40 μg mL^−1^) and PEG-b-PAsp-g-CA4-FITC (on the FITC basis) for 2,4 and 6 h. The cells were then trypsinized and uptake-analyzed by the MACSQuant flow cytometer. Intracellular distribution was assessed by confocal microscopy following DAPI staining 4 h after incubation.

### Pharmacokinetics

Pharmacokinetics was characterized by measuring CA4 levels by collecting the eye arterial blood of SD female rats (about 250 g). Specifically, six rats were divided into two groups of 3 each, and they were injected with free CA4P (50.0 mg kg^−1^on the basis of CA4) or PEG-b-PAsp-g-CA4 (50.0 mg kg^−1^ based on CA4) via tail veins, respectively. Eye arterial blood was collected at predetermined times (2, 5, 15, 30, 60, 90, 120, 240, 360, 480 min) and was heparinized and centrifuged to obtain plasma. Next, 50μL of the plasma sample was deproteinized with 250μL of acetonitrile [contains α-naphthylamine (40 μg mL^−1^)], vortexed for 5 min, and centrifuged at 10,000 rpm for 10 min. The supernatant was filtered by using 0.22 μm filters before HPLC measurement. The pharmacokinetics assay of PEG-b-PAsp-g-CA4 and CA4P was performed by fluorescence detector HPLC (FLD-HPLC) with a fluorescence detector at 290 nm Ex and 380 nm Em, and the elution was performed with methanol and water (v/ v = 7: 3) pumped at a flow velocity of 1.0 mL min^−1^ at 40 °C.

### In vivo imaging

To explore the tumor-targeting capability of CA4-NPs in vivo, Hepa1-6-bearing mice were i.v. injected with Cy5.5-loaded nano drugs followed by near-infrared optical imaging. Briefly, mice were subcutaneously inoculated with Hepa1-6 cells (1 × 10^7^ cells) in the right flank. When tumor reached 150–200 mm^3^, mice were randomly divided into two groups (free Cy5.5 and PEG-b-PAsp-g-CA4-Cy5.5) at an identical 50 μg kg^−1^ Cy5.5. 2,6, 12 and 24 h after injection, NIR fluorescent images were captured by in vivo imaging system (Tanon ABL X6, China). Finally, mice were sacrificed and heart, liver, spleen, lung, kidneys and tumor of each mouse were harvested for more detailed comparison.

### Tissue biodistribution study

To explore the tissue biodistribution study further, mice were subcutaneously inoculated with Hepa1-6 cells (1 × 10^7^ cells) in the right flank. When tumor reached 150–200 mm^3^, mice were randomly divided into two groups (free CA4P and PEG-b-PAsp-g-CA4) via the tail vein at dose of 40 mg CA4/kg. At 24 h, the mice were killed and their tumors, hearts, livers, spleens, lungs, kidneys were collected. Tissue samples were washed in ice-cold saline blotted with paper towels to remove excess fluid and weighed. Then the tumors and main organs were homogenized in water (1 g tissue per 1 mL) to get suspensions. And 100 μL tissue samples were deproteinized into 300 μL of acetonitrile by vortex for 5 min, and centrifuged at 10,000 rpm for 10 min. The test conditions of tissue biodistribution study was performed the same as pharmacokinetics assay.

### Immunofluorescence staining assay

The expressions of CD31, HIF-1α, PD-L1, CD4, CD8, LAG-3, TIM-3 were analyzed by immunofluorescence staining assay. The tumor tissues were excised and fixed in 4% buffered paraformaldehyde for 24 h, then embedded in paraffin and sliced to a thickness of 5.0 μm. The tumor tissue slides were deparaffinized by xylene, hydrated through a gradient of alcohol, boiled in 0.01 M sodium citrate buffer (pH 6.0) for antigen retrieval. After treating in blocking medium (5% BSA PBS buffer) for 20 min, the tumor tissue slices were first incubated with a monoclonal primary antibody (CD31, HIF-1α, PD-L1 CD4, CD8, LAG-3, TIM-3), and incubated with a fluorescently labeled secondary antibody (Cy3/AF488/AF594), then imaged by confocal laser scanning microscopy (CLSM).

### Tumor models

Healthy female C57BL/6 mice (20 ± 2 g) were purchased from Charles River (Beijing), and handling procedures were according to the guidelines of the Regional Ethics Committee for Animal Experiments of China Pharmaceutical University. Hepa1-6 tumor-bearing mice were selected as the animal model to assess the anti-tumor effect. Hepa1-6 cells were harvested after cell culture. Then, cells were suspended in PBS (5 × 10^7^ cells mL^−1^, 200 µL) and subcutaneously injected near the mammary region of mice.

### In vivo antitumor efficacy

When the tumor volume reached about 100 mm^3^, the nude mice were randomly divided into 6 groups with 6 mice in one group. Then the mice were administrated with PBS (group 1), CA4-NPs (20 mg kg^−1^) (group 2), CA4-NPs (40 mg kg^−1^) (group 3), aPD-L1 (group 4), CA4-NPs (20 mg kg^−1^) + aPD-L1 (group 5), and CA4-NPs (40 mg kg^−1^) + aPD-L1(group 6). The ratio of CA4 amount and body weight was about 5 mg kg^−1^ or 10 mg kg^−1^. The dosage of aPD-L1 was 50 µL for each mouse. The tumor size and body weight were recorded every day during the treatment. The mice were sacrificed after 15 days, and then the tumors and major organs were isolated and stained by hematoxylin & eosin (H&E). Besides, Ki67 and TUNEL immunostaining were also applied for all the tumors. Finally, the survival time of the remaining mice for each group were monitored additionally until day 60 and the survival curves were produced.

### Measurements of cytokines

To evaluate the level of IFN-γ and IL-4, frozen tumors were homogenized in PBS solution at 4 °C. All homogenized samples were centrifuged and the supernatants were stored at − 80 °C before analysis. Meanwhile, the blood samples were centrifuged to obtain the serum. Then cytokines both in mice serum and tumor were analyzed with corresponding ELISA kits (R&D Systems, Minneapolis, Minnesota, USA) according to the manufacturers’ instruction.

### Statistical analysis

Data were calculated and processed as mean ± SD. Comparison between groups was analyzed with Student’s t-test or one-way analysis of variance (ANOVA). Statistical differences were considered significant at *p < 0.05, **p < 0.01, ***p < 0.001, ****p < 0.001.

## Supplementary Information

**Additional file 1:** **Figure S1.** Images of the excised tumors after different treatments; shown are PBS-treated mice and mice receiving after injection with CA4-NPs (20 mg kg^-1^), CA4-NPs (40 mg kg^-1^), aPD-L1, CA4-NPs (20 mg kg^-1^) + aPD-L1, CA4-NPs (40 mg kg^-1^) + aPD-L1, CA4P (40 mg kg^-1^), n=10.** Figure S2 **Tissue distributions of CA4 in mice receiving free CA4P and PEG-b-PAsp-g-CA4 at 24 h.** Figure S3** ^1^H NMR spectra of BLA-NCA.

## Data Availability

All data generated or analyzed during this study are included in this published article.
