# Supplementary material for: Co-administration of combretastatin A4 nanoparticles and anti-PD-L1 for synergistic therapy of hepatocellular carcinoma
Source: J Nanobiotechnology. 2021 May 1;19:124. doi: 10.1186/s12951-021-00865-w (PMC8088584; doi:10.1186/s12951-021-00865-w)
Supplement: Supplementary file 1 — Additional file 1: Figure S1. Images of the excised tumors after different treatments; shown are PBS-treated mice and mice receiving after injection with CA4-NPs (20 mg kg-1), CA4-NPs (40 mg kg-1), aPD-L1, CA4-NPs (20 mg kg-1) + aPD-L1, CA4-NPs (40 mg kg-1) + aPD-L1, CA4P (40 mg kg-1), n=10. Figure S2 Tissue distributions of CA4 in mice receiving free CA4P and PEG-b-PAsp-g-CA4 at 24 h. Figure S3 1H NMR spectra of BLA-NCA. [file 12951_2021_865_MOESM1_ESM.docx]

**Additional Material**


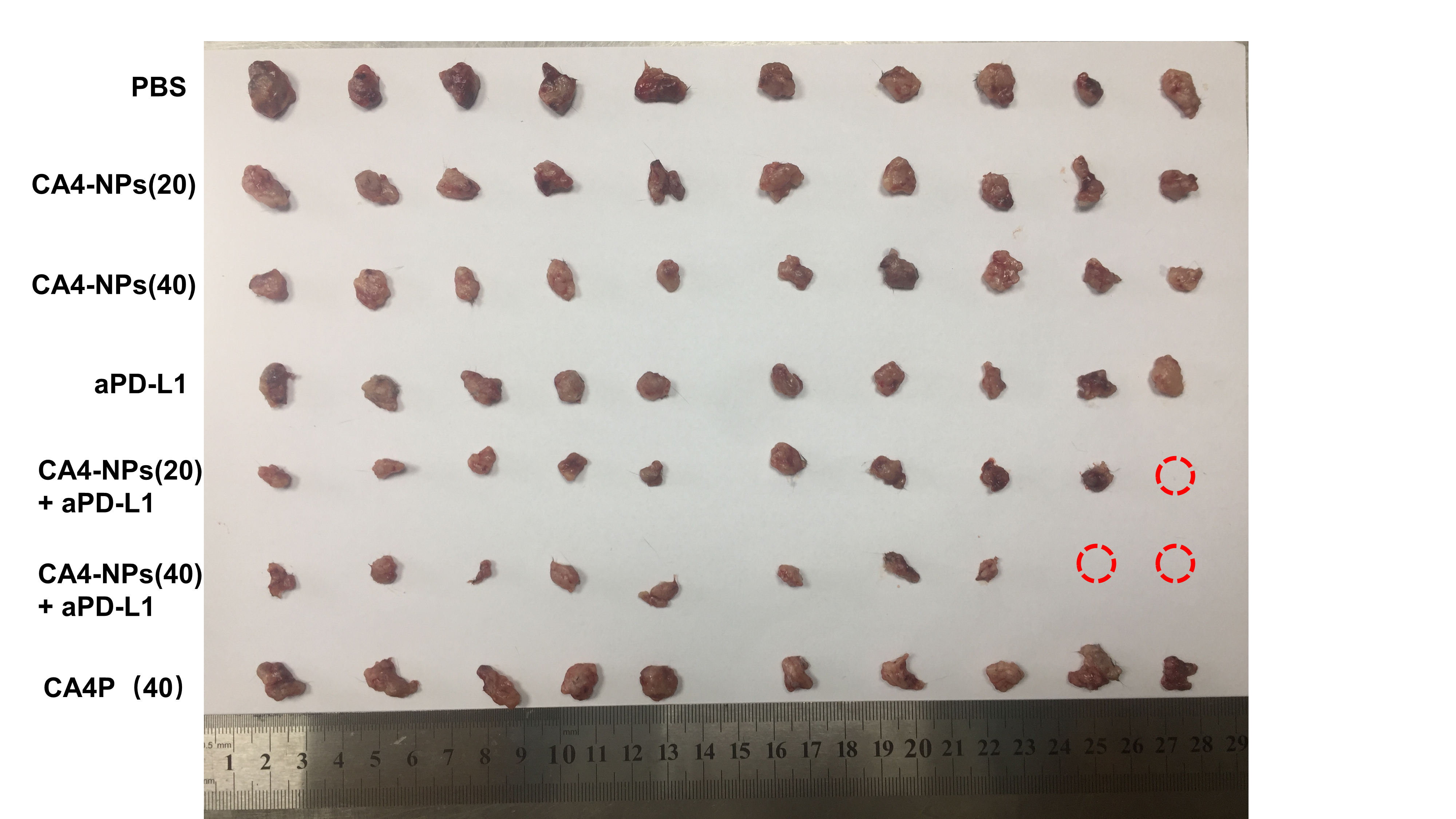


**Figure S1** Images of the excised tumors after different treatments; shown are PBS-treated mice and mice receiving after injection with CA4-NPs (20 mg kg^-1^), CA4-NPs (40 mg kg^-1^), aPD-L1, CA4-NPs (20 mg kg^-1^) + aPD-L1, CA4-NPs (40 mg kg^-1^) + aPD-L1, CA4P (40 mg kg^-1^), n=10.


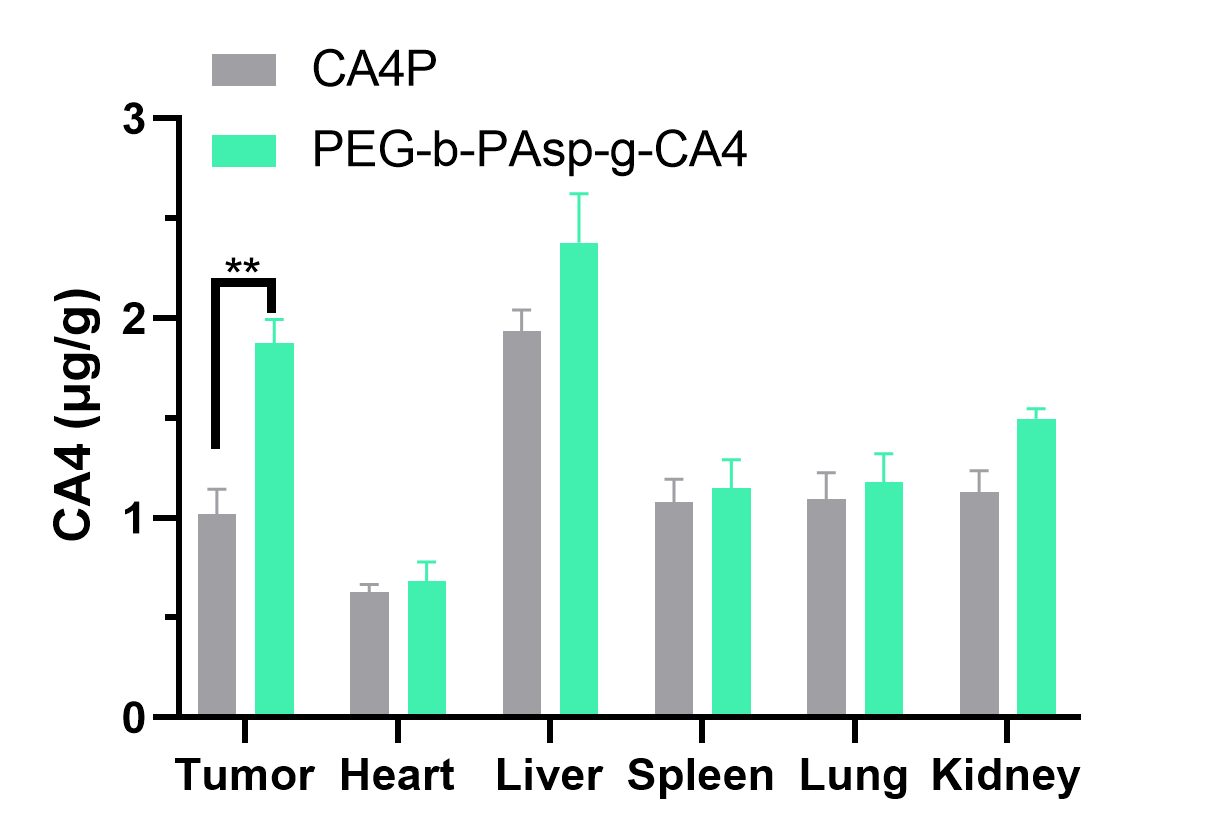


**Figure S2** Tissue distributions of CA4 in mice receiving free CA4P and PEG-b-PAsp-g-CA4 at 24 h.


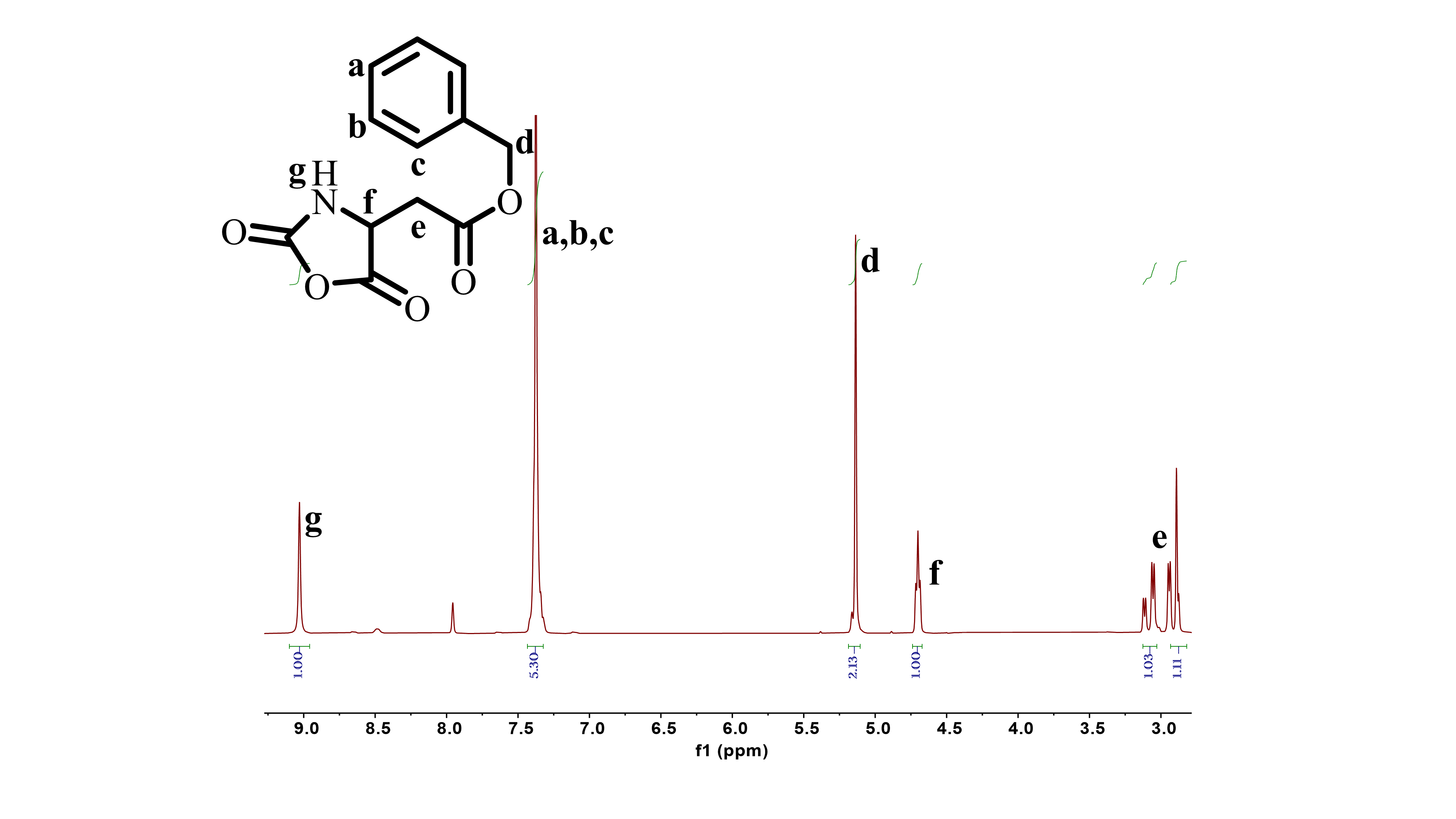


**Figure S3** ^1^H NMR spectra of BLA-NCA.
